# Supplementary material for: A model of tuberculosis clustering in low incidence countries reveals more transmission in the United Kingdom than the Netherlands between 2010 and 2015
Source: PLoS Comput Biol. 2020 Mar 27;16(3):e1007687. doi: 10.1371/journal.pcbi.1007687 (PMC7141699; doi:10.1371/journal.pcbi.1007687)
Supplement: S1 Text — Details of the models: S1.1) Posterior distributions for the model parameters; S1.2) Comparison between the Poisson lognormal model and the negative binomial distribution model fits for the UK and the NL; S1.3) Posterior distribution for the proportion of cases not due to recent transmission; S1.4) Posterior distribution for the reproduction number in the UK and the NL. (PDF) [file pcbi.1007687.s001.pdf]

Supplementary Information for ‘A model of tuberculosis clustering in low incidence countries reveals more transmission in the United Kingdom than the Netherlands between 2010 and 2015’

S1.1 Posterior distributions for the parameters from the Poisson Lognormal and Negative Binomial models

We simulated the generation of TB clusters using a mortal branching process model. Each cluster starts with an index case. The index case is joined by second epidemiologically-unrelated case infected with the identical genotype with probability p - this second case was either infected abroad or before the observation period. The index case also generates a number of secondary cases via direct transmission. The number of secondary cases is drawn from a distribution - either a Poisson lognormal distribution, or a negative binomial distribution. The process then repeats for all other cases, i.e. each case can be joined by an epidemiologically-unrelated case and generates a number of secondary epidemiologically-related cases. In the situation where all cases are infected abroad, then each case generates no secondary infections and no cases are due to recent transmission.

We fit the three parameters of the branching process model (one for probability of generating an epidemiologically-unrelated case and two for the distribution of secondary cases) using ABC-MCMC, implemented in the EasyABC R package. As described in the main paper, we used fifty logorarithmically-binned cluster sizes as the metrics and obtained 10,000 samples from the posterior distributions.

Figure A shows the posterior distributions for the branching process model with a Poisson lognormal distribution of secondary cases for the UK. Figure B shows the posterior distributions for the branching process model with a Poisson lognormal distribution of secondary cases for the NL. Figure C shows the posterior distributions for the branching process model with a negative binomial distribution of secondary cases for the UK. Figure D shows the posterior distributions for the branching process model with a negative binomial distribution of secondary cases for the NL.

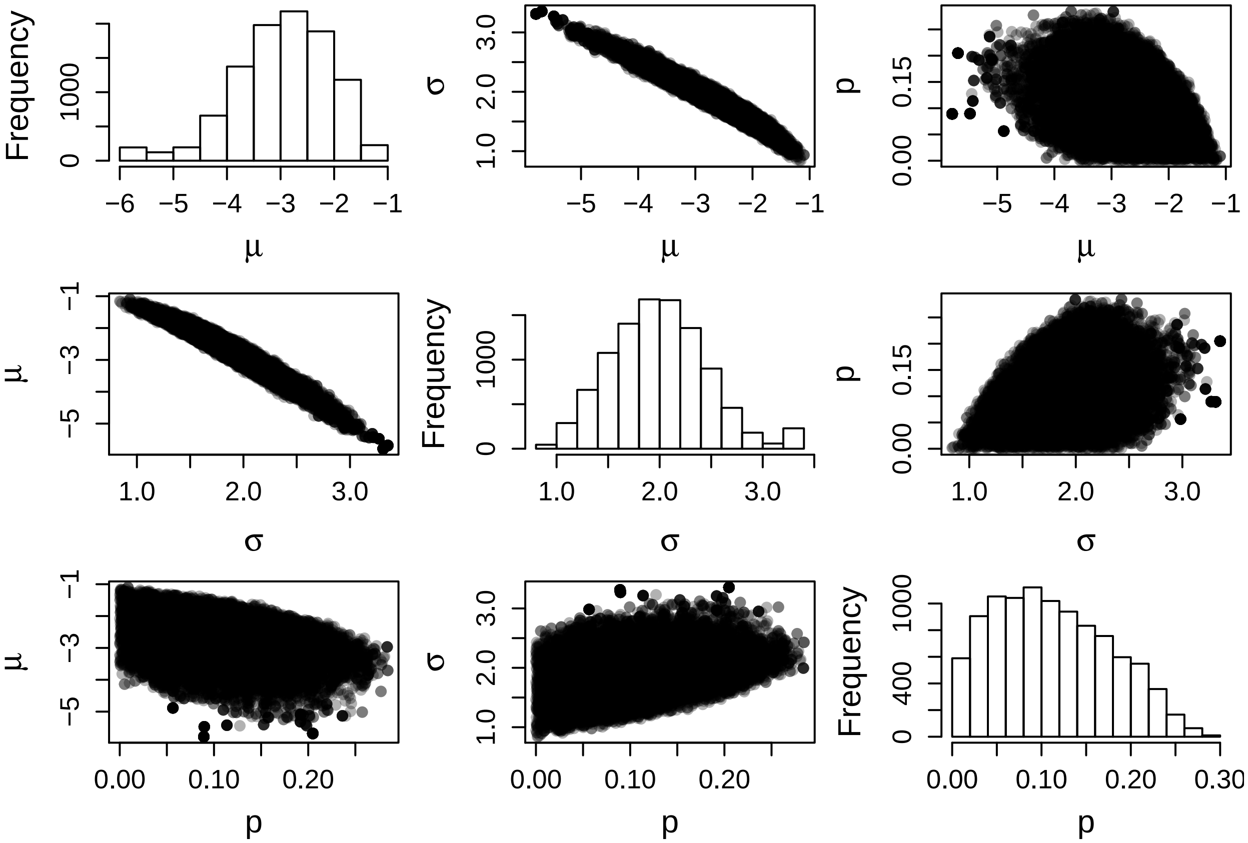

Figure A: Posterior distributions for the three parameters of the branching process model with a Poisson lognormal distribution (mean of the lognormal distribution, standard deviation of the lognormal distribution, and the overlap probability) for secondary within-country infections for the UK 2010-2015.

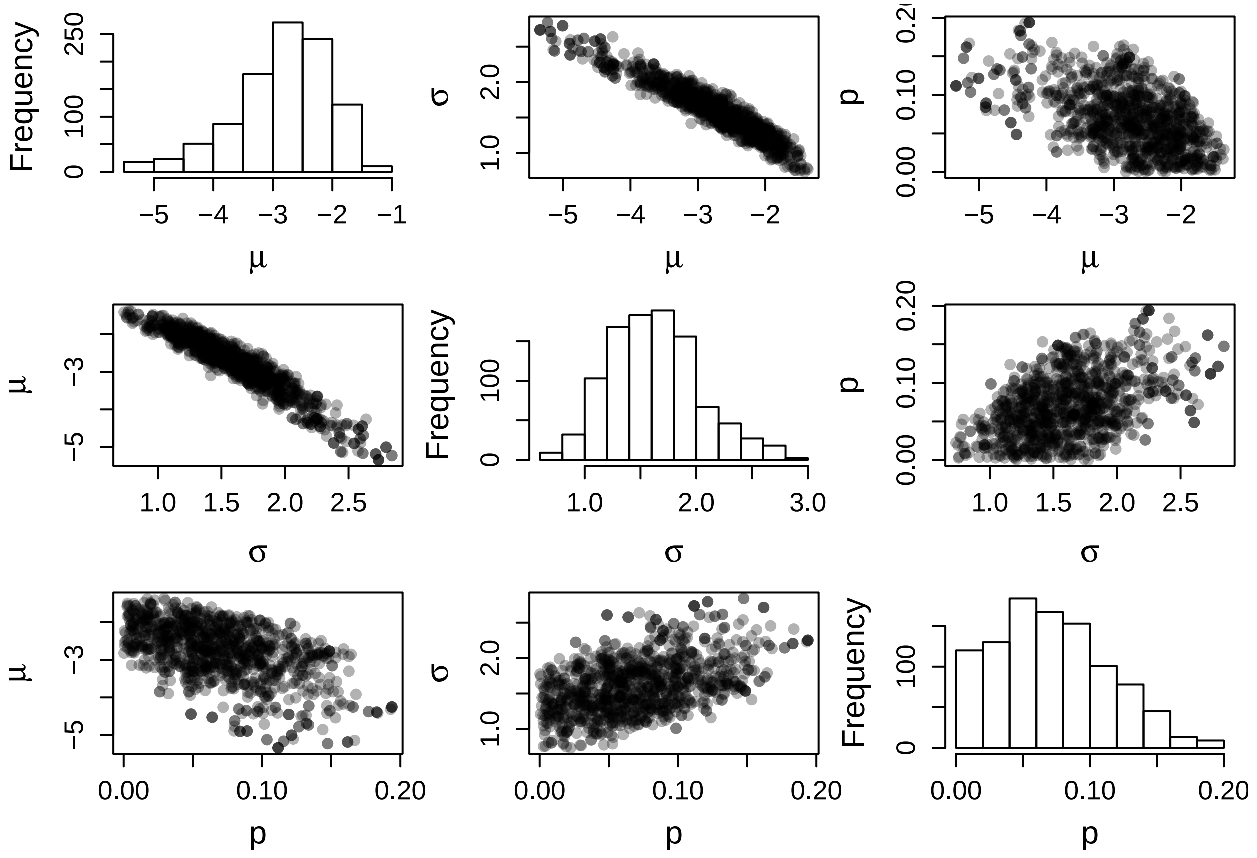

Figure B: Posterior distributions for the three parameters of the branching process model with a Poisson lognormal distribution (mean of the lognormal distribution, standard deviation of the lognormal distribution, and the overlap probability) for secondary within-country infections for the NL 2010-2015.

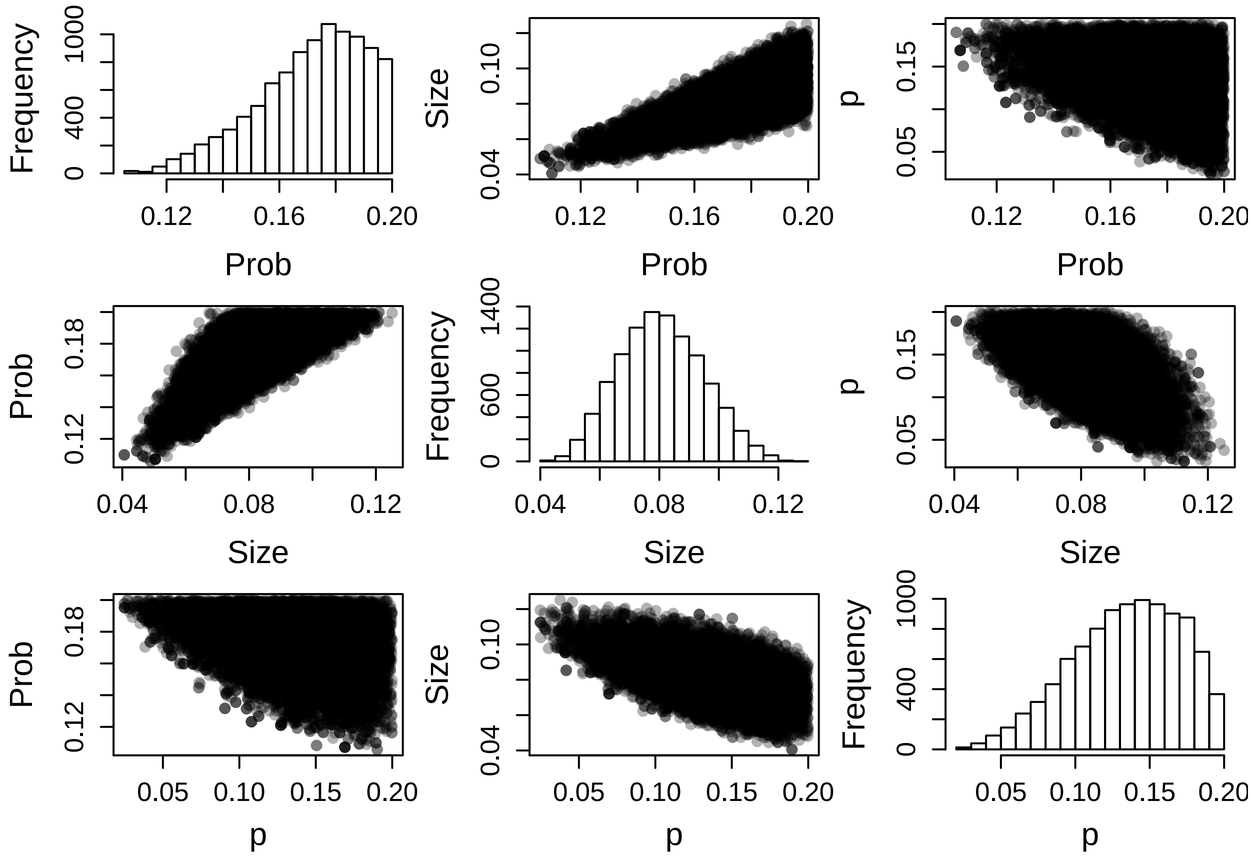

Figure C: Posterior distributions for the three parameters of the branching process model with a negative binomial distribution, and the overlap probability) for secondary within-country infections for the UK 2010-2015.

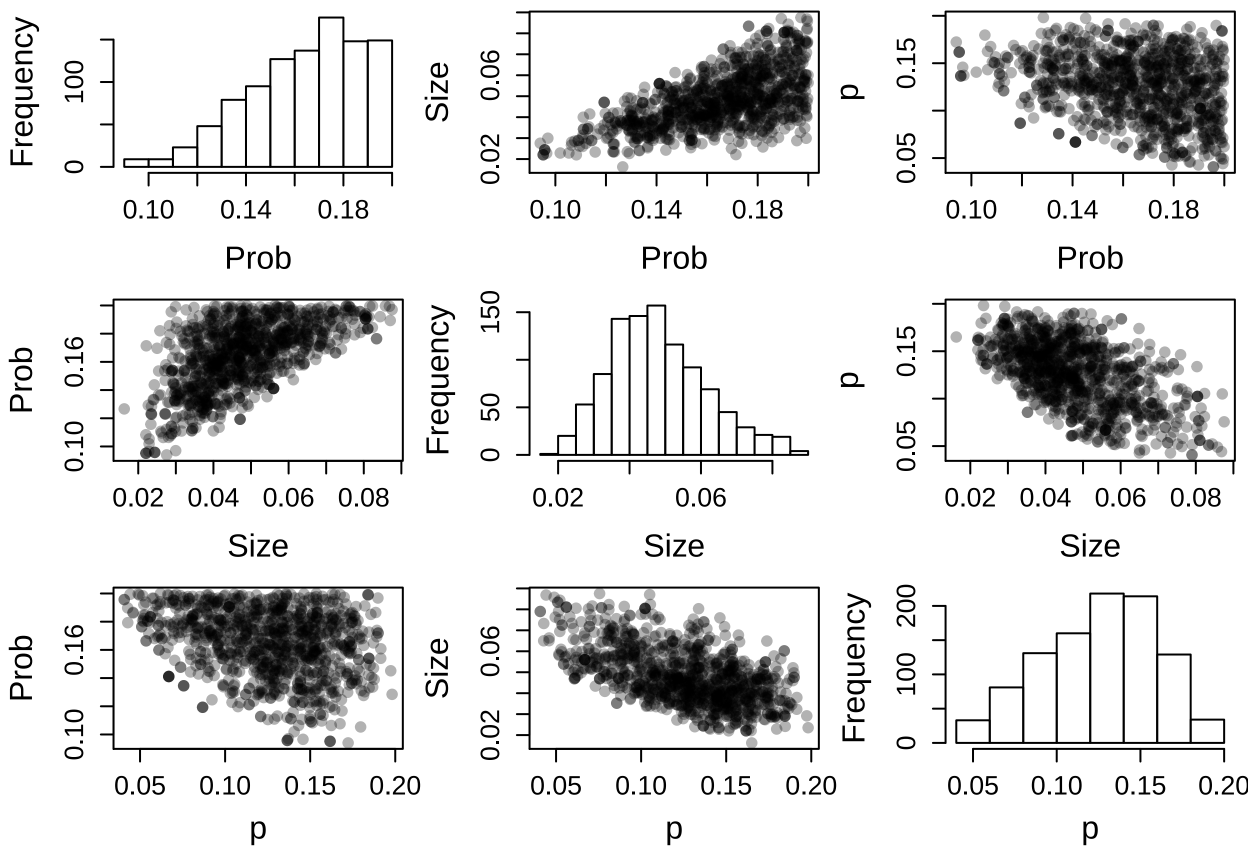

Figure D: Posterior distributions for the three parameters of the branching process model with a negative binomial distribution for secondary within-country infections for the NL 2010-2015.

S1.2 Comparison between the Poisson lognormal model and the Negative Binomial distribution model fits for the UK and the NL

We used two measures of cluster size distribution to assess model fit, proposed by Luciani et al.: the proportion of unmatched cases (i.e. clusters of size 1) and the number of unique cluster sizes.

In the main paper, we demonstrated that a branching process model with the negative binomial distribution for secondary cases was not able to capture the frequency of larger cluster sizes that is observed in the UK (figure 2, main paper). The model with a Poisson lognormal distribution of secondary cases captured both the frequency of clusters of size 1 and larger clusters. Both models were able to reproduce the cluster size distribution in the Netherlands.

The figures below (figs Ea and b, Fa and b) show the model simulations generated from 1,000 samples from the posterior distributions for the two models. Consistent with figure 2 in the main paper, the Poisson lognormal model results have a wider distribution and are more variable, compared to the negative binomial model.

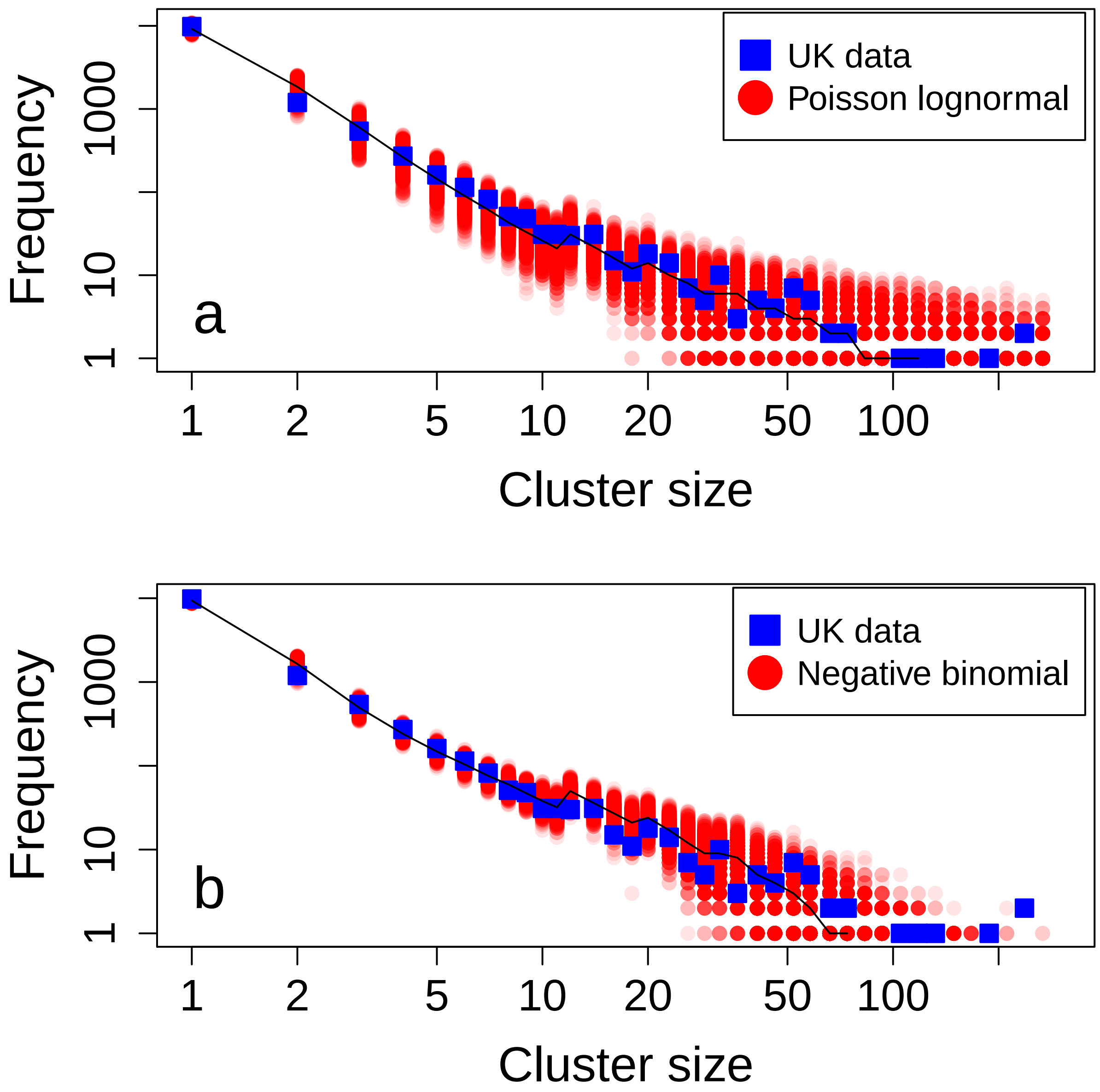

Figure E: Posterior distributions for the three parameters of the branching process model with a negative binomial distribution for secondary within-country infections for the UK 2010-2015.

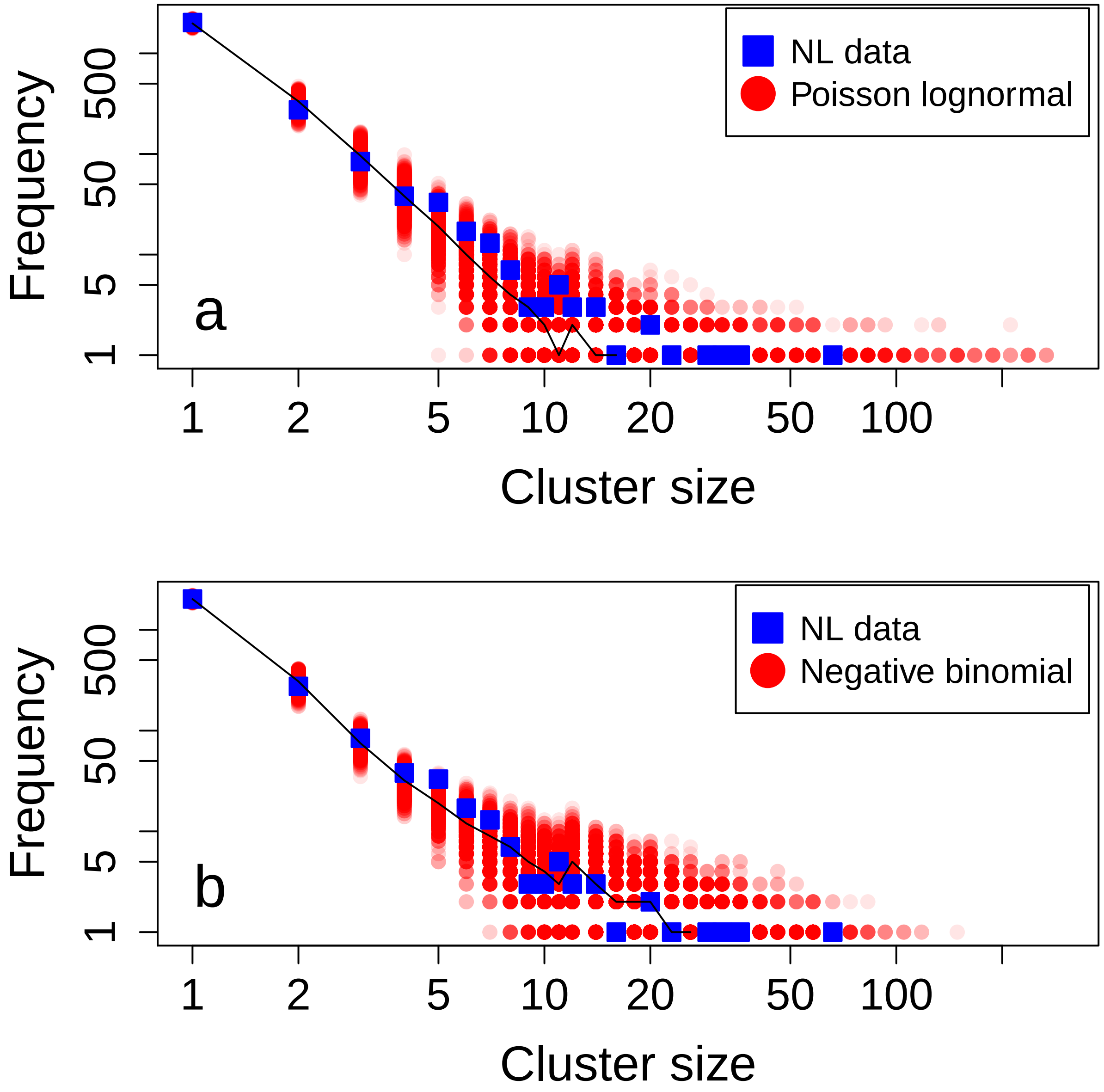

Figure F: The distribution of cluster sizes in the NL between 2010 and 2015 and the poisson lognormal model (a) and the negative binomial model (b).

S1.3 Posterior distribution for the proportion of cases not due to recent transmission

The origin of cases is estimated during model simulation by counting the number of cases generated via direct transmission (drawn from a Poisson lognormal distribution) and the number of epidemiologically-unrelated cases.

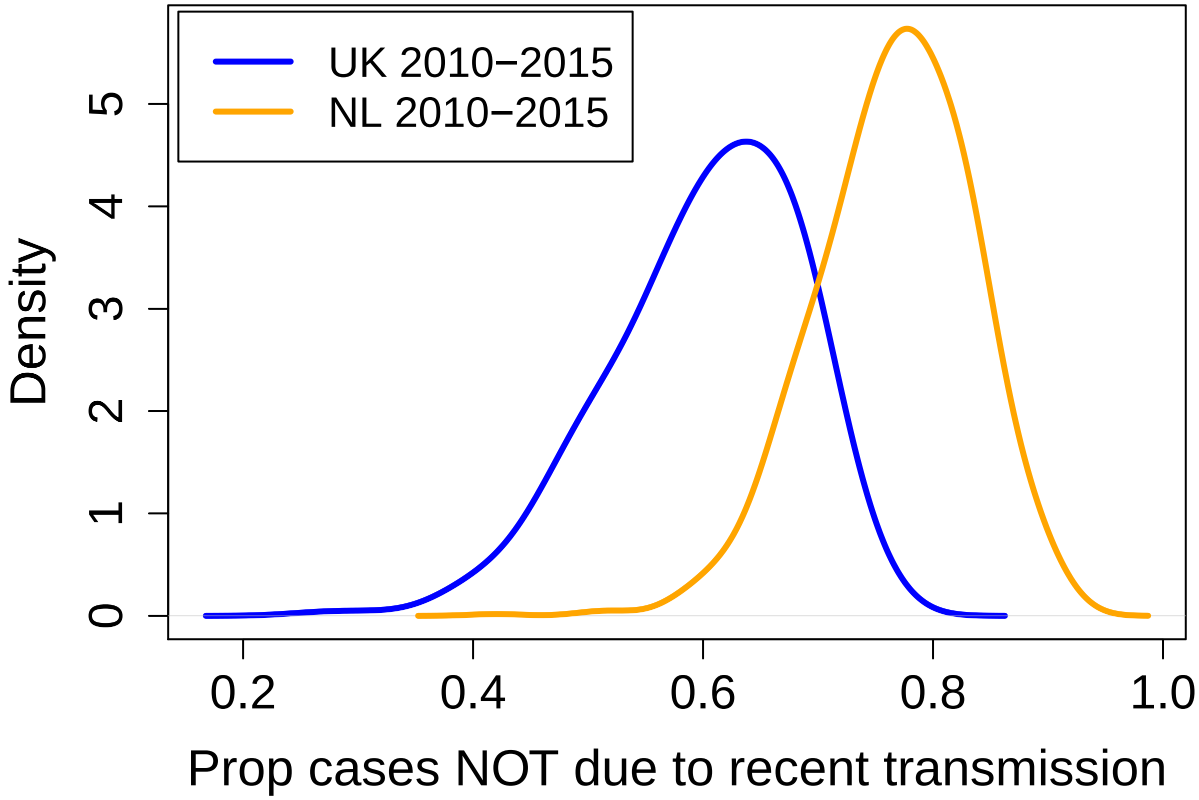

Figure G: The posterior distribution for the proportion of cases not due to recent transmission (i.e. due to importation or reactivation) in the UK and the NL 2010-2015.

S1.4 Posterior distribution for the reproduction number in the UK and the NL

The average reproduction number in the model comes from the distribution of secondary cases. The model is contrained to have $R < 1$, and this is supported by epidemiological evidence. The reproduction numbers are estimated using the posterior parameter values of the Poisson lognormal model as $R = \exp(\mu + \sigma^2/2)$.

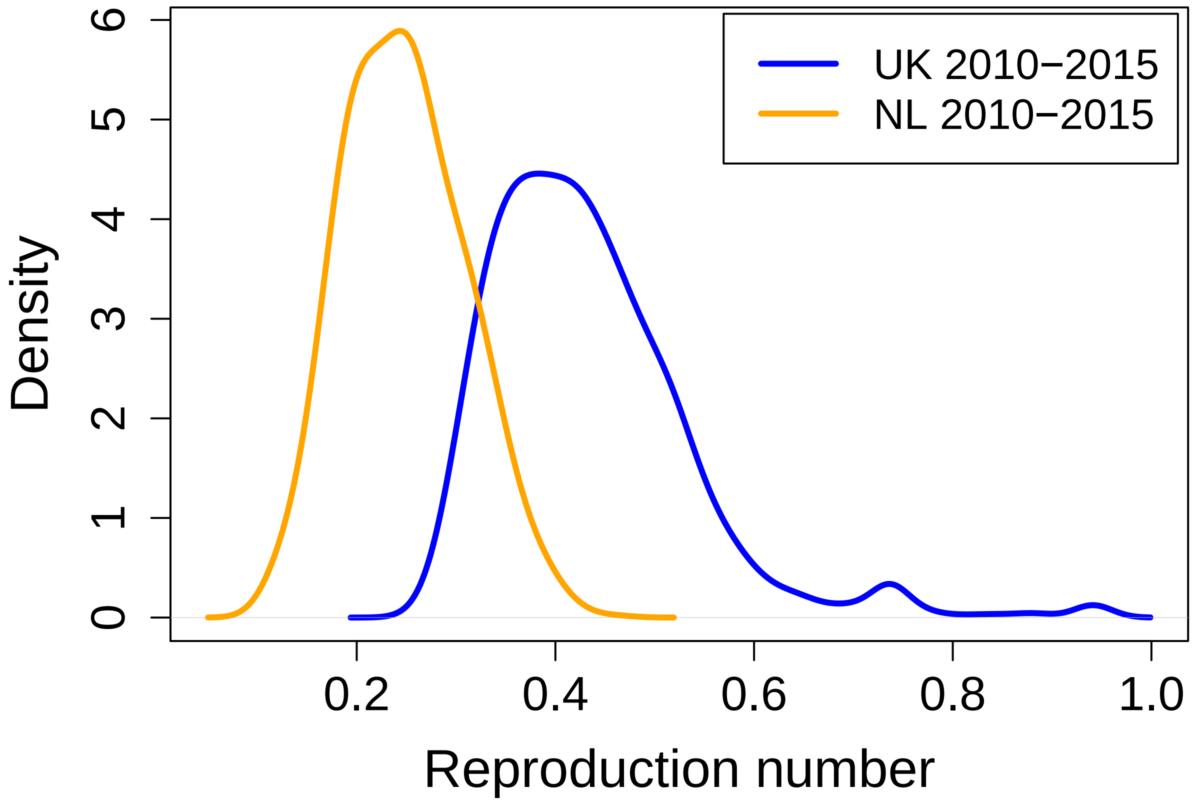

Figure H: The posterior distribution for the reproduction numbers in the UK and the NL 2010-2015.
