# Supplementary material for: A model of tuberculosis clustering in low incidence countries reveals more transmission in the United Kingdom than the Netherlands between 2010 and 2015
Source: PLoS Comput Biol. 2020 Mar 27;16(3):e1007687. doi: 10.1371/journal.pcbi.1007687 (PMC7141699; doi:10.1371/journal.pcbi.1007687)
Supplement: Attachment — Submitted filename: ReviewerComments.docx [file pcbi.1007687.s003.docx]

Ellen Brooks-Pollock

University of Bristol

[Ellen.Brooks-Pollock@bristol.ac.uk](mailto:Ellen.Brooks-Pollock@bristol.ac.uk)

Tel: (+44) 0117 3314064

3 November 2019

Dear Editors,

Please find enclosed our revised manuscript “*A model of tuberculosis clustering in low incidence countries reveals more transmission in the United Kingdom than the Netherlands between 2010 and 2015*”.

We thank the reviewers for their positive and thoughtful comments, which have helped improve the manuscript. The main changes are

- We have included a detailed discussion of model performance, focusing on the differences between the standard negative binomial assumption and the Poisson lognormal model. We feel this significantly adds to the paper as the metrics we now use can be readily compared to previous approaches and other situations.
- We have improved the precision of the language in reporting results.
- We have included more detail on the fitting process.

We include a point-by-point response to the reviewers’ comments below.

We hope that this manuscript is now acceptable for inclusion in the PLoS Computational Biology Special Issue on Mathematical Modelling of Infectious Disease Dynamics, and look forward to your response.

Your sincerely,

Ellen Brooks-Pollock, on behalf of the authors.

Point-by-point response to reviewers

Reviewer #1

Brooks-Pollock and colleagues present an analysis of TB genotyping data in the United Kingdom and the Netherlands using branching processes. The methods are interesting and well suited to the objectives. The result that improvements in TB prevention in the UK similar to what has been done in the Netherlands could lead to an important decrease of TB incidence is convincing and important. However, several points of the paper need to be improved before publication, in particular the lack of rigor regarding the use and reporting of statistical methods.

We thank the reviewer for their positive comments about the importance of this paper, and helpful suggestions. Below we aim to address the points they raise and convince them that the changes we have made improve the reporting and use of statistical methods.

Major points.

I have a problem with the way statistical inference and causality are handled in the results. First, the formulation suggests causality when only association is inferred (e.g. line 125: “having pulmonary disease increased the likelihood of belonging to a cluster”). In this example, the association could be explained by confounding factors, and even if causality exists it could go on the other direction, from belonging to a cluster to having pulmonary disease. The authors need to be a lot more careful with this very basic concept.

Thank you for pointing this out. As risk factors for clustering have been reported comprehensively elsewhere (including in a systematic review that we now reference, Fok et al. 2008), we decided to remove the short paragraph at the beginning of the results that reported on the relationship between cluster size and pulmonary status and birth country and focus instead on the mathematical model. For all the other results, we have taken care to appropriately interpret and report findings. We have amended the paragraphs at the beginning of the results section to:

“On average, 70% (42%, 98%) of clustered cases involved pulmonary disease, compared to 53% of all cases (Figure 1a). Between 2010 and 2015, 73% of cases in the UK were non-UK born. A lower proportion of those cases are diagnosed with pulmonary TB[19] (47%) compared to UK born cases (69%). Within a cluster, 64% (10%,95%) of cases are UK-born.”

“67% of all cases involved pulmonary disease, whereas within clusters, on average 70% (26%,100%) of cases were pulmonary (Figure 1b). As in the UK, non-NL born cases make up the majority, 69%. A lower proportion of non-NL born cases were diagnosed with pulmonary TB (63%) compared to NL born cases (76%). Within a cluster, 66% (33%,100%) of cases are non-NL born.”

Second, the authors need to choose a framework for statistical inference and stick to it. If a null hypothesis significance testing framework is chosen (as suggests the use of p values), then the authors should systematically report effect sizes, confidence intervals and exact p-values for each performed inferred relationship, and not for instance R-squared values (line 127), nothing at all (line 135) or only “p value >0.10” (line 139).

We now use a single statistical framework for reporting the results based on
Clauset, Shalizi & Newman (2009) “Power-law distributions in empirical data”. We use the Kolmogorov–Smirnov (KS) statistic to assess the power-law model fit with a hypothesis framework.

Third, I’m very surprised to see the authors interpret the absence of statistical significance as an absence of effect, especially since this common mistake got a lot of publicity a few months ago with the Nature paper by Amrhein, Greenland and McShane. The authors should reformulate their conclusions on lines 126 (“had no effect”), 138 (“no consistent relationship”) and 345 (“there was no association”).

Thanks – see our response above. We have refocused the paper on the mathematical model and taken care to avoid unwarranted interpretations of statistical significance.

Fourth, a visual inspection of the fit (which is not even shown) is not sufficient evidence to infer that a power law “describes well” the data (line 143) or that a Poisson-lognormal model “captures the entire distribution” better than a negative binomial model. The authors should use model selection tools (AIC, DIC, cross-validation…) to be able to support such claims. A plot comparing the fits of different models might also help convince the readers.

We thank the reviewer for this comment and have made a number of changes to address it, improving our manuscript in the process.

1. We have expanded on the figure in the supplementary information to show the equivalent fits for the two different models for both countries.
2. For the power-law fitting, we have used a hypothesis testing framework with a Kolmogorov-Smirnov statistic as proposed by Clauset et al. and implemented in the poweRlaw R package.
3. We have included a more detailed comparison of both models in terms of two metrics used by Luciani et al.: the number of clusters of size 1 and the number of cluster sizes that appear exactly once. These two metrics combined demonstrate that only the Poisson lognormal model is able to capture both ends of the cluster size distribution for the UK data. As the NL data contain fewer large clusters, both the negative binomial model and the poisson log-normal model capture the data well. We didn’t use a conventional model selection tool because the negative binomial model doesn’t capture the heavy tail of the UK data at all – the probability of observing the larger UK clusters given the negative binomial model was zero. This can be clearly seen in the new figure 2.

Minor points

- Abstract/intro: The given intervals should be systematically defined.

We have defined the intervals in the methods section and use that definition consistently.

- Line 87: I think the authors mean exactly the opposite.

Reference 10 demonstrated that, in fact, UK-born cases are more likely to belong to cluster of TB.

- Line 103: The authors need to define what recent transmission means earlier.

We now define this on line 103:

*“Here, we propose and develop a method to estimate the distribution in the number of secondary cases (the reproduction number) and the percentage of cases that are due to recent (since 2010), within-country transmission from the information in cluster size distributions for TB in the UK and the NL.”*

- Lines 103-204: The authors should make it clear that these results are predictions conditional on the chosen model.

We now explicitly indicate when we are using the model results as opposed to the description of the data.

- Line 107: The title of the paragraph is “cluster size distribution” but no distribution is shown beyond the proportion of clusters of size 1. I would suggest showing the histograms in Fig. 1, together with the fits as suggested before.

We have removed this section heading. We present the cluster size distributions in figure 3 (previously figure 2), along with the model predictions. We decided not to present the distributions earlier to avoid repetition.

- Line 115: No explanation is given regarding the time limits applied here.

This is due to data availability which we now make explicit in the results section.

- Line 184: Figure 3 is absolutely terrible and impossible to understand. The authors should definitely find a better way than pie charts to visualize the distribution of secondary cases.

We have replaced this figure with a different type of plot that hopefully makes the results easier to understand.

- Line 262: Including cases that were not genotyped wouldn’t alter the estimates of transmission only if these cases are missing at random. This should be discussed in the context of the UK and the NL.

We have removed this statement; the impact is potentially complex, as you suggest, and is not central to the argument of the paper.

- Line 396: Where does this definition of superspreading come from? If it was used in previous works a reference should be added.

Previous work has used various definitions of superspreading, including more than 6.2 secondary cases and as the tail of the distribution. Our use of 10 secondary cases is somewhat arbitrary and intended to be illustrative.

- Line 416: It is a bit clumsy to qualify approximate bayesian computation as an exact likelihood method.
We didn’t intend this interpretation and believe that confusion was caused by an unfortunate placement of two references. The sentence now reads:

*“In contrast to previous approaches that have used exact likelihood methods for fitting cluster size models to data[16,31], we use Approximate Bayesian Computation (ABC)[32,33].”*

Reviewer #2: This study analyses large data sets of clusters of tuberculosis based on MIRU-VNTR typing from the UK and from the Netherlands. In both places the incidence of TB is dropping so the effective reproduction number is below one. By fitting a model that includes the importation of new cases and a subcritical branching process to the data the authors are able to estimate the effective reproduction number and assess the predicted distribution of cluster sizes under the model. The focus is on pulmonary disease (the proportion of cases that are pulmonary is similar across countries and across cluster sizes). Fitting is performed with approximate Bayesian computation. The authors highlight the importance of superspreaders who transmit >10 cases each and discuss the epidemiological implications of reducing disease transmission.

This study makes excellent use of the large genetic data set; the model is simple yet able to reproduce features of the data successfully. A major issue, however, is that the manuscript feels incomplete at times - it's as if details are assumed to be obvious or already known or perhaps unimportant. Crucial details are missing particularly in the description of the simulation model and the ABC inference. I think it would be better to supply details especially since the current manuscript is not terribly long. Some examples are given below among specific comments and suggestions.

- Title: What is meant by "universal" in the title?
We intended to indicate that the model is able to describe data at all scales involved, but have now removed it from the title to avoid confusion.

- Luciani et al 2008, Infection, Genetics and Evolution, considered the distribution of TB cluster sizes using population models. It could be worth looking at as part of the background information.
Thank you for reminding us of this paper. Re-reading it has proved useful for our analysis. We used the two properties of cluster size distributions that Luciani et al. describe (the number of singletons and number of cluster sizes that appear exactly once) to compare the performance of the negative binomial model and the Poisson lognormal model – see the new figure 2.

- L 251. I appreciate the argument that WGS alone may not provide enough information to estimate reproduction numbers, but this sentence is a bit unclear. I would explain why in terms of genetic markers and mutation rates.

We have reworded this to:

“With sufficient data, WGS can be used to re-construct transmission trees and directly estimate reproduction numbers[30], however in many cases WGS alone is not sufficient, and needs to be combined with epidemiological data and statistical inference[31].”

- L 268 "However previous analysis found that right censoring..." This sentence is vague and cryptic -- spell out what the problem is. Right censoring of what? Why did it not affect the overall results? What is the meaning of "overall" results?

We have rewritten this paragraph:

“Further, we assumed that clusters were fully observed. In reality, only culture-confirmed cases can be genotyped and there may still be on-going transmission within a cluster. In 2015, genotyped cases represented 60.1% of all cases in the UK and 67% of all cases in the NL. The data are right censored because clusters may not have run their full course; this will apply particularly to strains that appeared for the first time towards the end of the datasets.”

- L 290 This sentence mentions "at least 23 loci" before defining the 24-locus MIRU-VNTR typing. I would define VNTR first for clarity.
Thank you, we have changed this.

- L 354 m is the result of a binomial distribution; p is given but what is the "n" (number of trials) parameter? At first I thought it might be prevalence but I saw that later it is the quantity C(X), though I found that confusing as I'll explain below.
The reason that n (the number of trials) is C(X) (the size of the cluster) is because we generate the cluster iteratively. For each case in the cluster, we draw a number of secondary cases resulting from direct transmission, and we also draw a random number to decide whether another unrelated case but infected with the same strain will join the cluster. Therefore, this is a binomial distribution with parameters C(X) and p. We have expanded the model explanation to make this clearer.

- L 359 "(see results)" Be more specific; say "see Figure 1" if that's what is meant.
We have now separated the methods and results and therefore have removed this statement.

- L 367 I find this equation unintuitive in the way it defines C(x) recursively. I can see that in a subcritical branching process the sum of r_i can go up to the total number of cases, but I don't understand the binomial term. How is the expression used computationally when the binomial term requires C(x) which it also contributes to? Could you please clarify and/or give details of how the expression is actually used in the simulation?
As mentioned above, we have expanded and reworded this section to make it clearer, including listing the steps of the branching process in pseudo-code.

- L384 Use of the Poisson-lognormal. This is fair enough but provide a reference from the ecological literature, e.g. Bulmer 1974, Biometrics.
We have added this reference.

- L395 Eqn 2. Where does this come from? Explain or give a reference. I would also add nearby the condition that R<1.

We added that equation 2 is the sum of a geometric series under the assumption that R<1.

- L 396 Is a superspreader one who generates more than 10 cases under the same model? Or has a different underlying rate? I believe it's the former but please clarify.
That is correct. We have clarified this in the text.

- L 415 The paragraph about the application of ABC is too brief in my opinion. I don't even know what parameters are being estimated. E.g. is p an unknown parameter to be estimated or set to some value? Give details of the prior distributions for them and the rationale for the choices. What's being estimated and what is fixed and assumed to be known? What distance metrics are used?

We have expanded this section to be explicit about what parameters we are estimating, prior constraints, the summary statistics and the methods for assessing goodness-of-fit. It now reads:

“We estimated three model parameters: two for the distribution of secondary cases (either negative binomial or Poisson log-normal) and one for the importation rate. We imposed prior constraints that all parameters are greater than zero and that the reproduction number, $R<1$. The target summary statistics were the number of observed clusters of a given size, logarithmically binned for a fixed number of bins. Using logarithmic binning attempts to compensate for the larger number of data points for lower cluster sizes. For $N$ bins, covering a range of cluster sizes from 1 to $C_{max}$, each bin is of length $\max\left( 1,\exp\left( n {\log C_{max}}/N \right) \right)$ for $n=1,\ldots,C_{max}$. We chose 50 bins, and the maximum cluster size was 300. For each set of proposal parameters, we simulated the model and binned the resulting clusters sizes in the same way as the data. The distance between the model and the data was calculated using the Euclidean distance:

$$\sum_{i=1}^{nbins} \sqrt{\left( D_{i}-M_{i} \right)^{2}},$$

where $D_{i}$ is the number of observed clusters in the $i$th bin and $M_{i}$ is the number of clusters in the $i$th bin as predicted by the model.”

What do the posterior distributions of the parameters look like? Would be good to see them along with credible intervals, as they are the values that generate the model predictions in Figure 2.

We have now included all posterior distributions in the supplementary information.

I assume the "95% CIs" given in L170-204 are the credible intervals from the posteriors. If so, make that clearer. Since there are multiple ways to compute credible intervals, how are they actually computed here? "CI" is often read as "Confidence interval" which is a frequentist concept and presumably not what is meant here; clarify what you mean.
We have now clarified that we are reporting credible intervals. We now state at the end of the methods section that credible intervals are calculated from the 2.5^th^ and 97.5^th^ quantiles from the posterior distributions.

- Fig 1 The panel labels seem to be missing. On the pulmonary axis, perhaps add "UK" and "NL" somewhere.
We have added them.
- Fig 2. Missing panel labels again.
We have added them.
- Fig 3. The pie chart is not visually helpful. There is a large sector and many small sectors. The scrunched up sectors have to be expanded to show another unhelpful pie. Could you find another way to show the data? A histogram or a table perhaps?
 We have changed figure 3.

**Have all data underlying the figures and results presented in the manuscript been provided?**
Large-scale datasets should be made available via a public repository as described in the *PLOS Computational Biology* [data availability policy](http://journals.plos.org/ploscompbiol/s/data-availability), and numerical data that underlies graphs or summary statistics should be provided in spreadsheet form as supporting information.

Reviewer #1: None

Reviewer #2: Yes
