# Supplementary material for: A model of tuberculosis clustering in low incidence countries reveals more transmission in the United Kingdom than the Netherlands between 2010 and 2015
Source: PLoS Comput Biol. 2020 Mar 27;16(3):e1007687. doi: 10.1371/journal.pcbi.1007687 (PMC7141699; doi:10.1371/journal.pcbi.1007687)
Supplement: Attachment — Submitted filename: ReviewerComments.docx [file pcbi.1007687.s004.docx]

Dear Editors,

Thank you for returning our manuscript with additional comments from reviewer 2. Their main suggestion was to revise the technical description of the model, which we have done. We provide a detailed description below, together with a highlighted version of the text.

We think these changes have improved the clarity of the manuscript and we hope that it is now acceptable for publication.

Yours sincerely,

Ellen Brooks-Pollock, on behalf of the authors.

Reviewer #1: I am satisfied with the revision.

Reviewer #2: The manuscript is improved and most of my suggestions/queries have been incorporate/addressed.

However, I would like to see the description of the model further developed near lines 354-365. The description of the branching process model is better but needs more work. The dummy variable i is used in a confusing way. First, it should be incremented somewhere - otherwise it stays at i=1 permanently and the loop can't be exited. Second, all cases become "relabelled" with respect to i after each "round" but this is not made explicit. Third, the value i=1 starts as the index case but after the first round i=1 is no longer the index case.

Thank you for these suggestions. We have substantially revised this section, as described below.

The equation on L364 still seems odd and misleading to me. As written, C(x) depends on itself but actually, this is a recursive function which is evaluated iteratively as the algorithm shows. This means that the C(x) on the left hand side is not actually the same quantity as the C(x) on the right hand side. After each round the C(x) must be updated.

We have removed the formula for the total cluster size C(x) to avoid confusion and because we feel the revised pseudo-code provides the necessary detail. Additionally, the code can be scrutinized via github (https://github.com/ldanon/TBclustr)

I think the iterative structure of the calculations should be made more explicit. My understanding of the algorithm (and the accompanying equation) is as follows.

As described in the manuscript r_i is the number of secondary cases per case, distributed as specified by the model. Define C(X,j) to be the total number of cases of genotype X after j iterations and M(X,j) to be the number of imported cases (of genotype X) after j iterations.

1. Set initial conditions:
j = 0,
C(X,0) = 1 and r_i = 1 when j = 0 (the index case),
M(X,0) = 0 (no imports until the outbreak has begun)

2. Compute

M(X,j) ~ Binomial(C(X,j), p)
C(X,j+1) = \sum_{i=1}^{C(X,j)} r_i + M(X,j)

3. Assign C(X,j) := C(X,j+1) and increment j to j+1

4. Repeat the recursion (steps 2 and 3) until C(X,j+1) = 0.

Thanks for this suggestion which has helped us improve this section. We have reexamined the way we describe the process and now it reads as follows:

*The branching process steps are as follows:*

1. *Start with the index case of a new cluster. Create a list of cases,* $L$ *containing a single case,* $L=\{1\}$*, such that the number of cases,* $n=1$*);*
2. *For each case* $i\in L$*, draw the number of secondary cases produced by* $i$*,* $r_{i}$*, from the relevant distribution (Poisson lognormal or negative binomial) and add* $r_{i}$ *cases to the end of the case list, such that* $n=n+r_{i}$*;*
3. *Draw a random number between 0 and 1; if this is less than probability* $p$*, generate an imported case and add it to the end of the case list, such that* $n=n+1$*;*

*Repeat steps 2 and 3 for all cases in* $L$*.*

Minor comments.

L267 comma instead of full-stop/period in front of "thereby"

Thanks - added.

L276 reword by moving clause "such as contract tracing" next to "Control policies"

Reworded as suggested.

L420 Are all the priors uniform (or "flat")? Whatever they are, supply the information.

Thanks, yes the priors are uniform - we have added this.

L421 I'm assuming that R>0 is also a condition so that 0<r<1.
</r<1.

Added this.
